# Supplementary material for: Study on the Bioactive Constituent and Mineral Elements of the Tibetan Medicine E’seguo from Different Regions of Ganzi Prefecture, China
Source: Molecules. 2024 Sep 1;29(17):4154. doi: 10.3390/molecules29174154 (PMC11397417; doi:10.3390/molecules29174154)
Supplement: Supplementary file 1 [file molecules-29-04154-s001.zip › molecules-3161039-supplementary.pdf]

The supplementary material is as follows:

1. Methodological investigation of total polysaccharides

1.1 Preparation of glucose standard curves

Preparation of reference substance solution: An appropriate amount of anhydrous glucose reference substance was precisely weighed, and then added to water to make glucose standard solutions containing 0.1246, 0.2076, 0.2906, 0.4152, and 0.4982mg per 1mL, respectively. The color was developed according to method 2.2. The blank control was 1.0mL ultra-pure water, and the concentration of the reference substance was taken as the horizontal axis. Absorbance was plotted as the ordinate and a standard curve was plotted. The results are shown in Table S1 below. The standard curve equation of glucose is y=1.0917x+0.0024, R^2=0.9997, indicating that there is a good linear relationship between the concentration range of glucose from 0.1246 to 0.4982mg·mL^{-1}.

Table S1 Glucose standard curve

Concentration （mg/ml）	0.1246	0.2076	0.2906	0.4152	0.4982
Absorbance (A)	0.136	0.229	0.323	0.458	0.543

1.2 Methodology investigation

1.2.1 Precision inspection

Six samples (1.0mL each) of the same sample extract were sucked into the test tube, and the color development was determined according to the method of item 2.2. The measured results are shown in Table S2, the average content of total polysaccharide is 27.34%, and the RSD is 0.88%, indicating that the method has good precision.

Table S2 Precision investigation

Number	1	2	3	4	5	6
Total polysaccharide （%）	27.54	27.04	27.61	27.40	27.18	27.25

1.2.2 Investigation of stability

Precision suction test sample extract 1.0mL placed in a test tube, according to the "2.2" operation for color determination, in 0h~3.0h every half an hour to measure the absorbance, the results are shown in Table S3, the results showed that the polysaccharide content decreased with the extension of time, within 3h sample solution is stable, RSD is 0.73%

Table S3 Investigation of stability

Time（h）	0	0.5	1	1.5	2	2.5	3
Total polysaccharide （%）	27.36	27.22	27.15	27.01	26.94	26.94	26.79

1.2.3 Investigation of stability

Six samples of the same sample were weighed with precision, each with 0.5g. The

color was developed and the absorbance was measured according to the item "2.2". The measured results are shown in Table S4, and the RSD is 1.43%, indicating that the method has good repeatability.

Table S4 Precision investigation

Number	1	2	3	4	5	6
Total polysaccharide （%）	28.23	28.04	27.29	27.53	27.38	27.36

1.3 Sample recovery rate investigation

A certain amount of glucose control solution was added to the test extract of the known content of Russeau fruit (the total polysaccharide content is %), then the color was developed and the absorbance was measured, and the average recovery rate of glucose was finally determined. The results are shown in Table S5. The average recovery rate of glucose is 99.91%, and the RSD is 1.3%, indicating that this method is accurate and good.

Table S5 Sample recovery rate investigation

Number	Weight of sample （g）	Content of sample （mg）	Amount added （mg）	Measured value （mg）	Sample recovery rate（%）	Average （%）	RSD （%）
1	0.2505	66.76	65.035	131.01	98.33	99.91	1.32
2	0.2502	66.68	65.035	130.51	98.20		
3	0.2504	66.73	65.035	132.12	100.53		
4	0.2505	66.76	65.035	132.50	101.06		
5	0.2504	66.73	65.035	131.33	99.35		
6	0.2503	666.70	65.035	132.74	101.50		

2. Methodological investigation of total polyphenols

2.1 Gallic acid standard curve

Preparation of reference substance solution: An appropriate amount of gallic acid reference substance was precisely weighed and added to water to make gallic acid standard solutions containing 0.05, 0.06, 0.07, 0.08, 0.09, and 0.1mg per 1mL, respectively. The color was developed according to the method of "2.2". The blank control was 1.0mL ultrapure water, and the concentration of the reference substance was used as the abscordinate and the absorbance as the ordinate. A standard curve was drawn. The results are shown in Table S6. The standard curve equation of gallic acid is $y=7.8429x+0.0863$, $R^2=0.9969$, indicating that there is a good linear relationship between the concentration of gallic acid in the range of 0.05-0.1 $mg{\cdot}mL^{-1}$.

Table S6 Gallic acid standard curve

Concentration （mg/ml）	0.05	0.06	0.07	0.08	0.09	1
Absorbance (A)	0.478	0.546	0.647	0.721	0.788	0.867

2.2 Methodology investigation

2.2.1 Precision inspection

Six samples (1.0mL each) of the same sample extract were preciously sucked into the test tube, extracted and developed color according to the method under "2.2", and the absorbance was measured. The measured results are shown in Table S7, with an average polyphenol content of 2.25% and an RSD of 1.30%, indicating good precision of this method.

Table S7 Precision investigation

Number	1	2	3	4	5	6
Total polyphenol content（%）	2.23	2.29	2.26	2.28	2.21	2.26

2.2.2 Investigation of stability

The 1.0mL extract of the test sample was placed in a test tube, extracted and developed according to the method under "2.2", and the absorbance was measured at 0.5, 1, 1.5, 2, 2.5, and 3h, respectively. The results are shown in Table S8. The results show that the polyphenol content decreases with the time, and the sample solution is stable within 3h, and the RSD is 2.21%.

Table S8 Stability investigation

Number	1	2	3	4	5	6
Total polyphenol content（%）	2.29	2.26	2.22	2.19	2.17	2.16

2.2.3 Repeatability test

Six samples (1.0g each) were weighed and extracted according to the method under "2.2", and the absorbance was measured. The measured results are shown in Table S9, with an average polyphenol content of 2.20% and an RSD of 2.58%, indicating good repeatability of this method.

Table S9 Repeatability investigation

Number	1	2	3	4	5	6
Total polyphenol content（%）	2.22	2.20	2.22	2.23	2.16	2.15

2.3 Sample recovery rate investigation

The known content of Russeka (total polyphenols content 2.19%) test extract was added with a certain amount of gallic acid control solution, mixed, color and measured the absorbance, and finally determined the average recovery rate of gallic acid. The results are shown in Table S10. The average recovery rate of gallic acid is 101.83%, and the RSD is 2.89%, indicating that this method is accurate and good.

Table S10 Sample recovery investigation

Number	Weight of sample （g）	Content of sample （mg）	Amount added （mg）	Actually measured values （mg）	Sample recovery rate（%）	Average （%）	RSD （%）
1	0.5019	11.09	10.84	22.38	104.06	101.83	2.89
2	0.5020	11.09	10.84	21.591	96.92		
3	0.5020	11.09	10.84	22.43	104.5		
4	0.5020	11.09	10.84	22.34	103.66		
5	0.5021	11.10	10.84	21.92	99.88		
6	0.5021	11.10	10.84	22.16	101.99		

3. Methodological investigation of L-malic acid

3.1 Repeatability test

Six samples of the same sample were prepared in parallel, and injected according to the chromatographic conditions under "2.3". The peak area of L-malic acid was determined and the concentration was calculated. The RSD of L-malic acid content was 2.02%, less than 3%, indicating good reproducibility of the method; the results are shown in Table S11.

Table S11 Repeatability investigation

Number	1	2	3	4	5	6	RSD
L-malic acid Content（%）	4.46	4.61	4.55	4.39	4.39	4.62	2.02

3.2 Precision inspection

The same sample solution was injected 6 times continuously according to the chromatographic conditions under "2.3" to determine the peak area of L-malic acid and calculate the concentration. The RSD of L-malic acid content was 1.81%, which was less than 3%, indicating good precision of the instrument; the results are shown in Table S12.

Table S12 Precision investigation

Number	1	2	3	4	5	6	RSD
L-malic acid Content（%）	4.69	4.66	4.66	4.87	4.65	4.66	1.81

3.3 Investigation of stability

The same sample solution was injected at 0, 2, 4, 6, 12, and 24 h according to the chromatographic conditions under "2.3", and the peak area of L-malic acid was determined and the concentration was calculated. The RSD of L-malic acid content was 1.39% and less than 3%, indicating that the test solution was stable within 24h. The results are shown in

Table S13.

Table S13 investigation of stability

Injection time （h）	0	2	4	6	12	24	RSD
L-malic acid Content（%）	4.57	4.60	4.57	4.57	4.61	4.74	1.39

3.4 Sample recovery rate investigation

The known content of E′seguo test solution (L-malic acid: 4.66%) was added to 10mL 0.60mg/ ml L-malic acid control solution, mixed, injected, determined the peak area of L-malic acid and calculated the concentration, and finally determined the average recovery rate of L-malic acid. The average recovery of L-malic acid was 100.0%. The RSD of the sample recovery was 1.7%, indicating that this method was accurate and good; the results are shown in Table S14.

Table S14 Sample recovery test

Number	Weight of sample （g）	Content of sample （mg）	Amount added （mg）	Actually measured values （mg）	Sample recovery rate（%）	Average sample recovery （%）	RSD ,（%）
1	0.1531	6.83	6.8	13.45	97.43	97.83	0.69
2	0.1531	6.83	6.8	13.44	97.21		
3	0.1533	6.84	6.8	13.48	97.66		
4	0.1531	6.83	6.8	13.45	97.33		
5	0.1538	6.86	6.8	13.58	98.80		
6	0.1536	6.85	6.8	13.55	98.52		

4. Methodological investigation of AA-2 β G

4.1 Repeatability test

Six samples of the same sample were prepared in parallel, and injected according to the chromatographic conditions under "2.3". The peak areas of the six components were determined and the concentrations were calculated. The RSD of AA-2 β G content was

0.87%, less than 3%, indicating good reproducibility of the method; the results are shown in Table S15.

Table S15 Repeatability investigation

Number	1	2	3	4	5	6	RSD
AA-2 β G content（%）	1.23	1.23	1.21	1.23	1.23	1.24	0.87

4.2 Precision inspection

The same sample solution was injected 6 times continuously according to the chromatographic conditions under "2.3", and the peak areas of the 6 components were determined and the concentrations were calculated. The RSD of AA-2 β G content was 1.16%, which was less than 3%, indicating good precision of the instrument; the results are shown in Table S16.

Table S16 Precision investigation

Number	1	2	3	4	5	6	RSD
AA-2 β G content（%）	1.24	1.24	1.23	1.23	1.23	1.20	1.16

4.3 Investigation of stability

The same sample solution was taken and injected at 0, 2, 4, 6, 12, and 24 h according to the chromatographic conditions under "2.2.4.1". The peak areas of the six components were determined and the concentrations were calculated. The RSD of AA-2 β G content was 0.87%, less than 3%, indicating that the test solution was stable within 24h; the results are shown in Table S17.

Table S17 Stability investigation

Injection time (h)（h）	0	2	4	6	12	24	RSD
AA-2 β G content（%）	1.23	1.23	1.23	1.20	1.23	1.23	0.87

4.4 Sample recovery rate investigation

Test solution of E′seguo fructus with known content (AA-2 β G: 1.2%), add 3mg/mlAA-2 β G control solution 0.61ml, mix, inject, determine the peak area of AA-2 β G and calculate the concentration, finally determine the average recovery of AA-2 β G. The average recovery of AA-2 β G was 100.0%. The RSD of the sample recovery was 1.7%, indicating that this method was accurate and good; the results are shown in Table S18.

Table S18 Sample recovery test

Number	Weight of sample（g）	Content sample（mg）	of Amount added（mg）	Actually measured values（mg）	Sample recovery rate（%）	Average sample recovery（%）	RSD ，（%）
1	0.1536	1.8739	1.8300	3.6851	98.9724		
2	0.1540	1.8788	1.8300	3.7086	99.9905		
3	0.1537	1.8751	1.8300	3.7446	102.1540	100.0	1.7
4	0.1533	1.8703	1.8300	3.7341	101.8471		
5	0.1539	1.8776	1.8300	3.6718	98.0447		
6	0.1539	1.8776	1.8300	3.6907	99.0795		

5. **Raw data table**

Table S19 Raw data table(n=3)

	Total polysaccharide %	Total acid%	Sugar acid ratio	Total polyphenols%	AA-2 β G %	L-malic acid%	Kg/kg)	Ca(g/kg)	Zn(ug/kg）	Mg(ug/kg）	Cu(ug/kg）	Fe(ug/kg）	Se(ug/kg）	Mn(ug/kg）
Number	25.2	1.8	14.2	1.8	1.5	2.6	4.9	1.5	50.4	5.8	13.1	0.7	0.7	0.6
M1-1	25.2	1.8	14.2	1.8	1.5	2.6	4.9	1.5	50.4	5.8	13.1	0.7	0.7	0.6
M1-2	23.0	1.5	15.5	2.6	2.1	2.4	4.8	1.7	55.4	7.4	11.5	0.7	0.6	0.7
M1-3	28.5	2.0	14.1	2.2	1.5	3.1	4.9	1.7	57.7	6.5	8.8	1.1	0.8	0.6
M1-4	30.0	1.9	16.1	2.1	1.7	2.7	4.8	1.2	37.7	6.2	8.0	1.0	0.9	0.6
SD	3.2	0.2	1.0	0.3	0.3	0.3	0.1	0.2	8.9	0.7	2.4	0.2	0.1	0.0
Average	26.7	1.8	15.0	2.2	1.7	2.7	4.9	1.5	50.3	6.5	10.3	0.9	0.8	0.6
CV	0.1	0.1	0.1	0.1	0.2	0.1	0.0	0.1	0.2	0.1	0.2	0.2	0.1	0.1
M2-1	23.2	2.0	11.8	2.2	1.8	3.0	5.0	1.8	43.0	6.8	8.2	1.1	0.9	0.7
M2-2	21.3	1.4	15.6	2.1	1.5	2.2	4.9	1.6	51.5	7.5	10.8	0.8	1.1	0.5
M2-3	25.9	1.7	15.2	2.4	1.4	2.8	5.0	1.4	35.8	6.1	8.1	0.9	1.3	0.6
M2-4	22.5	1.6	14.5	2.7	1.9	2.3	4.9	1.4	35.3	6.5	10.6	0.9	0.9	0.6
SD	2.0	0.3	1.7	0.3	0.2	0.4	0.1	0.2	7.6	0.6	1.5	0.1	0.2	0.1
Average	23.2	1.6	14.3	2.3	1.6	2.6	5.0	1.5	41.4	6.7	9.4	0.9	1.1	0.6
CV	0.1	0.2	0.1	0.1	0.1	0.1	0.0	0.1	0.2	0.1	0.2	0.1	0.2	0.2
M3-1	25.2	2.9	8.8	2.9	1.7	3.8	4.8	1.8	57.2	7.1	10.1	1.0	0.8	0.6
M3-2	24.3	2.5	9.7	2.7	1.3	3.2	4.9	1.6	43.6	5.5	13.5	1.0	1.0	0.5
M3-3	21.1	2.3	9.1	2.0	1.2	3.8	5.2	1.3	53.9	4.9	10.1	0.9	1.3	0.5
M3-4	23.5	2.6	9.2	2.5	1.4	3.6	5.0	1.5	51.6	5.8	11.2	0.9	1.1	0.5
SD	1.8	0.2	0.4	0.4	0.2	0.3	0.2	0.2	5.8	0.9	1.6	0.0	0.2	0.0
Average	23.5	2.6	9.2	2.5	1.4	3.6	5.0	1.5	51.6	5.8	11.2	0.9	1.1	0.5

CV	0.1	0.1	0.0	0.2	0.1	0.1	0.0	0.1	0.1	0.2	0.1	0.0	0.2	0.1
M4-1	17.7	2.2	8.2	1.7	0.8	3.9	5.1	2.2	102.2	8.5	7.2	0.6	1.8	0.2
M4-2	16.4	2.0	8.3	1.3	0.8	3.8	5.1	2.3	90.0	7.7	6.4	0.7	1.9	0.3
M4-3	17.0	1.9	9.1	1.9	0.8	3.8	4.7	2.1	92.3	7.9	8.5	0.5	2.1	0.3
M4-4	17.5	1.8	9.8	1.7	0.8	3.1	3.9	2.0	82.4	10.2	8.8	0.8	2.2	0.3
SD	0.6	0.2	0.7	0.3	0.0	0.4	0.6	0.1	8.2	1.2	1.1	0.1	0.2	0.0
Average	17.1	2.0	8.8	1.6	0.8	3.6	4.7	2.2	91.7	8.6	7.7	0.7	2.0	0.3
CV	0.0	0.1	0.1	0.2	0.0	0.1	0.1	0.1	0.1	0.1	0.1	0.2	0.1	0.1
M5-1	25.9	1.0	27.0	1.7	1.0	1.6	3.9	1.6	39.1	9.8	5.2	1.2	0.7	0.5
M5-2	18.7	1.4	13.8	2.0	1.2	2.0	4.7	1.3	38.4	9.6	5.5	1.0	0.9	0.5
M5-3	28.1	1.2	23.4	1.6	0.9	2.0	4.9	1.5	48.8	8.3	6.0	1.0	0.8	0.4
M5-4	24.2	0.9	26.6	1.9	1.1	2.0	4.7	1.6	41.2	7.2	6.1	0.9	1.0	0.5
SD	4.0	0.2	6.2	0.2	0.1	0.2	0.4	0.2	4.8	1.2	0.4	0.1	0.1	0.1
Average	20.2	0.9	19.4	1.5	0.9	1.6	3.7	1.2	34.5	7.2	4.7	0.8	0.7	0.4
CV	0.2	0.2	0.3	0.1	0.2	0.1	0.1	0.1	0.1	0.2	0.1	0.1	0.2	0.1
M6-1	31.6	2.5	15.4	2.9	1.5	3.4	4.9	0.0	31.1	8.2	7.6	1.1	0.9	0.7
M6-2	35.5	1.9	18.6	2.7	1.6	3.3	5.4	1.7	37.1	6.5	10.8	0.8	1.2	0.7
M6-3	24.2	1.8	13.8	2.1	1.7	2.5	4.8	1.5	35.6	7.1	8.0	1.0	0.8	0.6
M6-4	38.5	2.2	13.6	2.6	1.3	3.6	5.0	1.7	44.2	7.1	10.7	1.0	0.9	0.5
SD	6.2	0.3	2.3	0.4	0.2	0.5	0.3	0.1	5.4	0.7	1.7	0.2	0.2	0.1
Average	32.4	2.1	15.4	2.6	1.5	3.2	5.0	1.6	37.0	7.2	9.3	1.0	1.0	0.6
CV	0.2	0.1	0.2	0.1	0.1	0.2	0.1	0.1	0.1	0.1	0.2	0.2	0.2	0.1
M7-1	25.4	2.8	9.0	2.6	1.5	3.8	4.7	1.3	47.8	5.5	10.4	0.8	1.1	0.5
M7-2	23.0	3.2	7.2	2.6	1.5	4.4	5.3	1.8	54.9	6.1	8.9	1.0	1.3	0.5
M7-3	22.4	2.8	8.2	2.0	1.4	4.2	5.0	1.4	44.9	5.6	8.5	1.0	1.1	0.6
M7-4	21.7	2.7	7.9	2.3	1.5	3.7	5.1	1.5	51.6	5.6	10.2	1.2	1.1	0.7
SD	1.6	0.2	0.8	0.3	0.1	0.3	0.2	0.2	4.4	0.3	0.9	0.1	0.1	0.1

Average	23.1	2.9	8.1	2.4	1.5	4.1	5.0	1.5	49.8	5.7	9.5	1.0	1.2	0.6
CV	0.1	0.1	0.1	0.1	0.0	0.1	0.0	0.1	0.1	0.0	0.1	0.1	0.1	0.2
M8-1	29.0	0.9	31.5	1.7	0.8	2.6	5.1	1.9	33.3	7.0	10.0	0.9	1.3	0.6
M8-2	26.5	0.8	31.7	1.6	0.8	2.6	4.8	1.7	40.7	6.6	9.2	0.8	1.1	0.6
M8-3	21.2	1.0	22.4	1.3	0.7	3.0	4.9	1.3	53.9	5.4	11.3	0.6	1.1	0.5
M8-4	24.2	0.8	29.9	1.9	0.9	2.0	4.7	1.6	54.8	7.0	8.2	0.8	1.4	0.5
SD	3.3	0.1	4.4	0.2	0.1	0.4	0.1	0.2	10.5	0.8	1.3	0.1	0.2	0.1
Average	25.2	0.9	28.9	1.6	0.8	2.6	4.9	1.6	45.6	6.5	9.7	0.8	1.3	0.6
CV	0.1	0.1	0.2	0.1	0.1	0.2	0.0	0.2	0.2	0.1	0.1	0.1	0.1	0.1
M9-1	28.2	2.6	11.1	2.5	1.4	3.2	5.2	1.3	68.5	5.4	4.2	1.4	1.5	0.7
M9-2	22.2	2.6	8.7	2.7	1.0	3.6	4.8	1.1	53.0	4.6	4.9	1.8	1.5	0.7
M9-3	25.5	2.6	9.8	2.4	0.9	3.4	5.2	1.0	60.3	4.7	5.3	2.0	1.1	0.7
M9-4	29.2	2.0	14.6	2.2	1.3	2.1	5.2	1.4	48.3	6.2	4.5	1.9	1.2	0.6
SD	3.1	0.3	2.6	0.2	0.2	0.7	0.2	0.2	8.8	0.7	0.5	0.3	0.2	0.0
Average	26.3	2.4	11.0	2.5	1.2	3.1	5.1	1.2	57.5	5.2	4.7	1.8	1.3	0.7
CV	0.1	0.1	0.2	0.1	0.2	0.2	0.0	0.2	0.2	0.1	0.1	0.2	0.2	0.1
M10-1	26.5	3.1	8.7	3.8	1.4	3.9	4.6	1.5	33.4	4.3	12.8	1.0	1.1	0.6
M10-2	25.1	3.3	7.5	3.5	1.7	4.6	5.0	1.4	34.5	5.7	10.7	0.7	1.2	0.5
M10-3	25.2	2.8	9.1	2.9	1.4	3.8	4.7	1.3	35.8	5.4	10.6	1.0	1.4	0.6
M10-4	25.4	3.1	8.3	3.1	1.5	4.1	5.2	1.7	42.5	5.4	10.3	0.8	1.4	0.6
SD	0.7	0.2	0.7	0.4	0.1	0.3	0.3	0.2	4.1	0.6	1.1	0.2	0.1	0.0
Average	25.6	3.1	8.4	3.3	1.5	4.1	4.9	1.5	36.5	5.2	11.1	0.9	1.3	0.6
CV	0.0	0.1	0.1	0.1	0.1	0.1	0.1	0.1	0.1	0.1	0.1	0.2	0.1	0.0
M11-1	24.7	2.6	9.4	2.5	0.8	2.8	4.7	1.5	58.9	5.9	9.4	0.8	1.0	0.5
M11-2	24.7	2.2	11.5	2.1	0.8	2.6	5.2	1.9	56.8	6.0	10.1	0.7	1.2	0.5
M11-3	26.9	2.7	10.1	2.1	1.1	3.4	4.7	1.7	61.2	5.5	13.1	0.6	1.3	0.5
M11-4	24.6	2.7	9.1	2.2	0.8	3.4	5.0	1.4	66.1	5.0	10.7	0.8	1.3	0.6

**SD**	**1.1**	**0.3**	**1.0**	**0.2**	**0.1**	**0.4**	**0.3**	**0.2**	**4.0**	**0.4**	**1.6**	**0.1**	**0.1**	**0.0**
**Average**	**25.2**	**2.5**	**10.0**	**2.2**	**0.9**	**3.1**	**4.9**	**1.6**	**60.8**	**5.6**	**10.8**	**0.8**	**1.2**	**0.5**
**CV**	**0.0**	**0.1**	**0.1**	**0.1**	**0.1**	**0.1**	**0.1**	**0.1**	**0.1**	**0.1**	**0.1**	**0.2**	**0.1**	**0.1**
**M12-1**	**22.6**	**3.0**	**7.5**	**2.6**	**1.3**	**4.0**	**5.2**	**1.5**	**79.6**	**6.0**	**7.6**	**1.3**	**1.4**	**0.4**
**M12-2**	**23.7**	**2.2**	**11.0**	**2.9**	**1.7**	**2.8**	**4.7**	**0.9**	**63.3**	**6.0**	**6.2**	**1.3**	**2.0**	**0.4**
**M12-3**	**27.0**	**2.4**	**11.5**	**2.8**	**1.7**	**3.6**	**4.9**	**1.5**	**77.9**	**6.8**	**5.5**	**1.2**	**2.0**	**0.4**
**M12-4**	**18.6**	**2.6**	**7.3**	**2.2**	**1.6**	**3.2**	**5.2**	**1.4**	**85.5**	**6.3**	**8.9**	**0.9**	**2.0**	**0.5**
**SD**	**3.4**	**0.4**	**2.2**	**0.3**	**0.2**	**0.5**	**0.2**	**0.3**	**9.5**	**0.4**	**1.5**	**0.2**	**0.3**	**0.1**
**Average**	**23.0**	**2.5**	**9.3**	**2.6**	**1.6**	**3.4**	**5.0**	**1.3**	**76.6**	**6.3**	**7.1**	**1.1**	**1.8**	**0.4**
